# Metformin anticancer: Reverses tumor hypoxia induced by bevacizumab and reduces the expression of cancer stem cell markers CD44/CD117 in human ovarian cancer SKOV3 cells

**DOI:** 10.3389/fphar.2022.955984

**Published:** 2022-08-15

**Authors:** Yuanchun Fan, Huimin Cheng, Yueping Liu, Shihao Liu, Scott Lowe, Yaru Li, Rachel Bentley, Bethany King, John Pocholo W. Tuason, Qin Zhou, Chenyu Sun, Hui Zhang

**Affiliations:** ^1^ The Department of Gynecology, The Fourth Hospital of Hebei Medical University, Shijiazhuang, China; ^2^ The Department of Pathology, The Fourth Hospital of Hebei Medical University, Shijiazhuang, China; ^3^ College of Osteopathic Medicine, Kansas City University, Kansas, MO, United States; ^4^ Internal Medicine, Swedish Hospital, Chicago, IL, United States; ^5^ Internal Medicine, MercyOne Des Moines Medical Center, Des Moines, IA, United States; ^6^ AMITA Health Saint Joseph Hospital Chicago, Chicago, IL, United States; ^7^ Radiation Oncology, Mayo Clinic, Rochester, MN, United States

**Keywords:** metformin, bevacizumab, CSCs, VEGF, HIF-1α

## Abstract

**Background:** The occurrence and development of solid tumors depend on the blood supply in the tumor microenvironment (TME). Blocking angiogenesis is a new therapeutic strategy to inhibit tumor growth. The anti-angiogenic drug bevacizumab has been approved for gynecological malignancies, especially for advanced recurring cervical cancers and recurring ovarian cancers (OC). Studies in OC have shown a limited effect of bevacizumab in the general population, with a slight improvement in progression-free survival (PFS) and no effect on overall survival (OS). This might be related to the bevacizumab’s role in aggravating the hypoxia in the TME, which helps maintain the stemness of ovarian cancer stem cells (CSCs) and promotes the invasion and metastasis of cancer cells. Drugs that target CSCs, such as metformin, may enhance the efficacy of anti-vascular therapies. Therefore, this study aimed to evaluate the effect of metformin combined with bevacizumab on the proliferation of OC cells both *in vitro* and *in vivo*, as well as on tumor hypoxia and tumor stem cell markers of human ovarian cancer SKOV3 cells.

**Methods:** The OC cell model SKOV3 was treated with metformin, bevacizumab, and cisplatin alone or in combinations. Cell Counting Kit-8 (CCK-8) was used to measure the rate of cell proliferation. Metformin and bevacizumab were studied *in vivo* in nude mice. SKOV3 cells were transplanted subcutaneously in nude mice, and different drug interventions were performed after tumor formation, including blank control, bevacizumab alone, metformin alone, cisplatin alone, bevacizumab + metformin, bevacizumab + cisplatin, metformin + cisplatin, and bevacizumab + metformin + cisplatin treatments. The growth of transplanted tumors was routinely monitored and visualized by the tumor growth curve. We used flow cytometry to examine the proportion of CD44^+^/CD117^+^ CSCs in each group. The immunohistochemistry (IHC) method was applied to detect expressions of vascular endothelial growth factor (VEGF), hypoxia-inducible factor 1α (HIF-1α), and microvascular density-associated factor CD34 in tumor cells. The limit dilution method was used to re-inject tumor cells in nude mice to examine the tumor recurrence rate.

**Results:** Combination therapy of metformin and bevacizumab significantly reduced the proliferation rate of SKOV3 cells and the growth rate of transplanted tumors in nude mice compared with the monotherapy effects. *In vivo* results showed that metformin significantly reduced the proportion of CD44^+^/CD117^+^ CSCs (*p* < 0.01). Although bevacizumab increased the proportion of CD44^+^/CD117^+^ CSCs, the addition of metformin did offset this fluctuating trend. The combination of bevacizumab, metformin, and cisplatin efficiently decreased the proportion of CSCs in the OC animal model. IHC results exhibited that expressions of VEGF, CD34, and HIF-1α in transplanted tumors were decreased by metformin alone compared with the control (*p* < 0.05). In the bevacizumab treatment, VEGF, and CD34 expressions were decreased, while that of HIF-1α was increased, suggesting that the degree of hypoxia was differentially aggravated after the bevacizumab treatment. The VEGF, CD34, and HIF-1α expressions in the bevacizumab + metformin + cisplatin group were the lowest among all other treatment groups (*p* < 0.05). Subcutaneous statistics of nude mice reseeded by the limit dilution method showed that the tumor recurrence rate in the bevacizumab + metformin + cisplatin group was relatively lower.

**Conclusion:** Metformin, bevacizumab combined with platinum-based chemotherapy can significantly inhibit the growth of ovarian cancer cells and transplanted tumors, which is due to the reduction of the proportion of CD44^+^/CD117^+^ CSCs and the alleviation of hypoxia in the tumor microenvironment. Therefore, this may be a reasonable and promising treatment regimen.

## Introduction

Among various gynecological malignancies, commonly occurring epithelial ovarian cancer (EOC) poses the most severe threat to women’s health. The absence of specific indicators for early diagnosis as well as the lack of effective treatments for the drug-resistant and relapsing EOCs relay to poor prognosis. Notably, 70% of patients with complete remission (CR) after the first line of treatment exhibit relapses within 3 years, and the 5-years survival rate of patients having the International Federation of Gynecology and Obstetrics (FIGO) staging of III-IV is only 30–40% (De Angelis et al., 2014). Moreover, even the recent advances in the tumor-targeted precision therapies for EOC have not been effective yet in improving the overall survival (OS) in these patients, especially for those with sudden relapse. It has been shown that the cancer stem cells (CSCs) play a central role in developing chemo-resistance as well as tumor recurrence in high-risk individuals (Prost et al., 2015; Yoshida et al., 2016). CSCs belong to a subset of stem cells primarily within the tumor microenvironment (TME) with an ability of unlimited self-renewal and multi- or pluripotency. Hypoxia provides a more conducive environment for the growth of CSCs via the induction of hypoxia-inducible factor-1 alpha (HIF-1α), while angiogenesis is crucial for the maintenance of stemness in CSCs (Zhang et al., 2021). In the TME, CSCs secrete a higher level of vascular endothelial growth factor (VEGF) compared to other types of cell populations. Increased VEGF levels facilitate the migration of endothelial cells to form new microvasculature in the TME, thus increasing the tumor microvasculature density (MVD). Therefore, targeting the TME, especially the angiogenesis and hypoxia-associated mechanisms, would be an effective therapeutic strategy to clinically control the proliferation and activity of CSCs in cancer progression and metastasis.

Recently, the monoclonal anti-VEGF antibody bevacizumab has been widely used in TME-targeted therapies to suppress angiogenesis in EOC patients. Real-world studies on ovarian cancer (OC) patients have shown that bevacizumab can significantly improve the progression-free survival (PFS) rate only in high-risk individuals, but not the OS (Burger et al., 2011; Perren et al., 2011; Oza et al., 2015). One of the possible explanations might be the proliferation and sequestration of CSCs through altered TME properties due to the anti-angiogenic therapy (Eyler and Rich, 2008). Therefore, new drugs and/or combination therapies should be explored further to tackle the challenge of cancer recurrence and sudden relapse as well as maximize the clinical outcomes and prognosis in OC patients. Metformin has demonstrated its efficacy in inhibiting the generation of CSCs (Hirsch et al., 2009; Bishnu et al., 2019) and improving hypoxic shocks in the TME (Li et al., 2019). Furthermore, studies have confirmed that metformin can target EOC-related CSCs in the TME, by limiting their proliferation and improving the sensitivity of cisplatin in combination therapy (Shank et al., 2012). Based on these findings, we speculated that the combination of metformin and bevacizumab might have synergistic effects on the treatment of OC.

Therefore, we tested different combined regimens of bevacizumab, metformin, and cisplatin to evaluate their efficacies in both *in vitro* and *in vivo* conditions. Additionally, we detected the expression of tumor stem cell marker CD44/CD117 in SKOV3 cells by flow cytometry. Examined the expressions of VEGF and HIF-1α, and CD-34 positivity for estimating the MVD in response to various treatment regimens by immunohisto-chemistry (IHC). The results of this study suggest a novel treatment strategy for OC in the preclinical stage and provide a reference for advanced stage EOC management.

## Materials and methods

### Cell lines

The ovarian cancer cell model SKOV3 was preserved and passaged in the pathology laboratory of the Institute of Oncology, the Fourth Hospital of Hebei Medical University. SKOV3 cells were cultured in McCoy′s 5A medium supplemented with 10% fetal bovine serum (FBS) and 1% penicillin-streptomycin (PS) in a humidified sterile incubator with 5% CO_2_ + 95% O_2_ at 37°C. The culture medium was changed every 24 h. Cells were passaged at about 80% confluency using 0.25% trypsin for subculture.

### Cell proliferation assay

After 24 h of culture, SKOV3 cells were seeded in 96-well plates (replicated in six wells per group) at a density of 1 × 10^3^ cells per well. Then, each group was treated with a pre-determined drug combination, followed by the CCK-8 assay. Briefly, 10 µL of CCK-8 reagent was added to each well and incubated for 2 h before measuring the optical density (OD) at 450 nm on a microplate reader. The OD value was proportional to the rate of cell proliferation. Cellular morphological changes were observed and photographed afterward.

### Animal experiments

The animal experiment protocol was approved by the Ethics Committee of The Fourth Hospital of Hebei Medical University and conducted by the National Institutes of Health (NIH) guidelines for the care and use of laboratory animals. Six-week-old female nude mice were obtained from Beijing Vito Lihua Laboratory Animal Technology Co., Ltd. (Beijing, China). SKOV3 cells (5 × 10^6^ per animal) were subcutaneously injected into the right hind limb, and the growth of the transplanted tumor was monitored daily. On the fifth day post-inoculation, mice with tumor diameters of 3–5 mm were randomly divided into eight treatment groups (*n* = 4): A) blank control, B) bevacizumab, C) metformin, D) cisplatin, E) bevacizumab + metformin, F) bevacizumab + cisplatin, G) metformin + cisplatin, and H) bevacizumab + metformin + cisplatin groups. All three drugs were intraperitoneally administered at the dose of bevacizumab-5 mg/kg/week, cisplatin-5 mg/kg/week, and metformin-250 mg/kg daily. The blank control group receive normal saline of the same volume

Tumor volume was measured every 2 days using the formula V = L (length) × W^2^ (width)/2. The diameter of transplanted tumor in nude mice was no more than 15 mm. The tumor growth curve was plotted by taking the mean of each group. Mice transplanted with SKOV3 cells were sacrificed in the fourth week after treatment. The mice anesthetized with 4% chloral hydrate 9 ml/kg were confirmed dead after cervical dislocation under deep anesthesia.

### Flow cytometry

The isolated xenograft tumors were cut into small pieces to prepare a single-cell suspension and adjusted to a density of 1 × 10^6^ cells/100 µL. Cells from each group were incubated with FITC-CD44 and PE-CD117 for 30 min for fluorescent labeling in the dark. Flow cytometry was performed on a FACS Aria machine (Beckman Coulter FC 500MPL, United States).

### Subcutaneous replantation of diluted cells in nude mice

The prepared cell suspensions were divided into two separate groups of 5000 cells and 10,000 cells in 100 µL of PBS and replanted to the right back of nude mice to examine the tumor formation rate.

### Hematoxylin and eosin (H&E) staining

Paraformaldehyde fixed tissue samples were cut into small pieces of 1 cm^3^ area and embedded in paraffin. A pathological microtome was used to slice the paraffin-embedded tissue blocks. The thickness of the coarse slice was 20μm, and that of the fine slice was 5 μm. The cut tissue was sequentially processed for spreading, dewaxing, immunostaining or antibody probing, drying, and finally sealing with neutral resin.

### Immunohistochemistry (IHC)

The excised tumor tissues were fixed in 10% neutral formalin solution overnight at room temperature, then paraffin-embedded and stained with respective antibodies. Anti-VEGF (Affinity, 1:100), anti-CD-34 (ABclonal, 1:100), and anti-HIF-1α (ABclonal, 1:100) antibodies were used for IHC staining. Four tumor tissues were selected from each treatment group, and three sections were cut from each tumor tissue block for IHC analysis. VEGF-positive and CD34-positive blood vessels and HIF-1α-positive cells were counted at 400x in each section.

### Statistical analysis

All statistical analyses were performed by SPSS 19.0 and GraphPad Prism 5.0 software tools. The experimental data are presented as mean ± standard deviation (SD). The differences between any two groups were calculated using an independent *t*-test. One-way analysis of variance (ANOVA) followed by a post-hoc multiple comparisons test was employed. A *p* < 0.05 was considered statistically significant.

## Results

### Effects of the combination of bevacizumab, metformin, and cisplatin on SKOV3 cell proliferation *in vitro*

**FIGURE 1:**
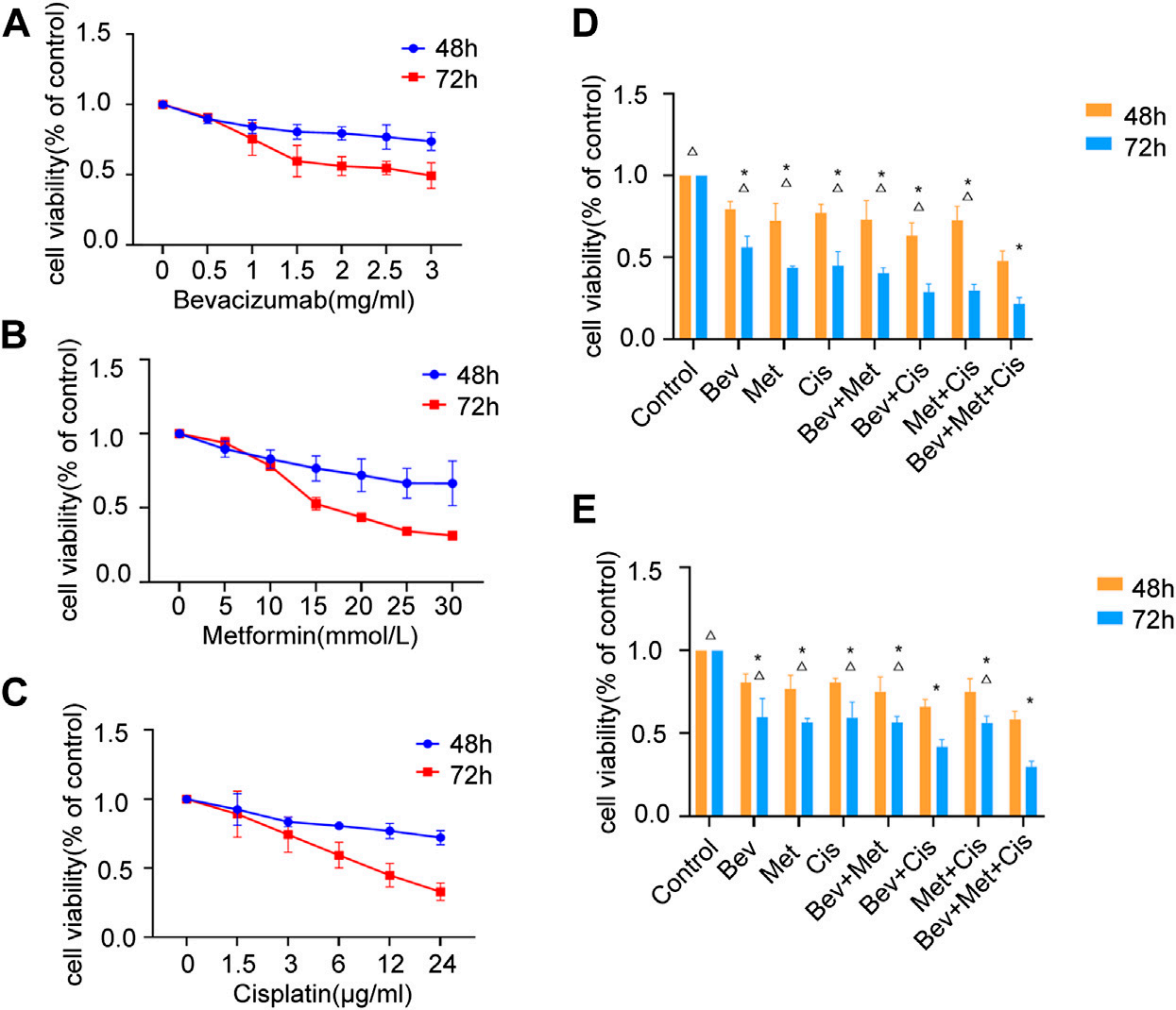
Effects of different drug combinations on SKOV3 cell proliferation *in vitro*. **(A)** Bevacizumab inhibited the proliferation of SKOV3 cells in doses ranging from 1 to 3 mg/ml. **(B)** Metformin reduced SKOV3 cells’ proliferation at the dose range of 10–30 mmol/L. **(C)** Cisplatin decreased the proliferation of SKOV3 cells in doses between 3 μg/ml and 24 μg/ml. **(D)** Effects of low-dose drug combination groups on the SKOV3 cell proliferation. **(E)** Effects of high-dose drug combination groups on the SKOV3 cell proliferation. Note: compared with control, *p < 0.05; compared with the three-drug combination group, △*p* < 0.05. Effects of different drug combinations on the morphology of SKOV3 cells.

To examine the effects of bevacizumab, metformin, and cisplatin on cancer cell growth, we treated SKOV3 cells with respective agents. The rate of inhibition of cell proliferation was gradually increased with the increasing dose of drugs. We found that these three drugs, individually and in combination, could significantly inhibit cell proliferation compared to the mock treatment (Figures 1A–C).

We set up different drug combinations, including low to high doses. The low dose regimen included 1.5 mg/ml of bevacizumab, 15 mmol/L of metformin, and 6 μg/ml of cisplatin. Compared with the corresponding blank controls at 48 and 72 h time points, the low dose group showed a statistically significant effect (*p* < 0.05), and the bevacizumab + cisplatin and bevacizumab + metformin + cisplatin groups had relatively higher efficacies compared with other groups (*p* < 0.05). There was no significant difference between these two groups (Figure 1D).

On the other hand, the high dose regimen was composed of 2 mg/ml of bevacizumab, 20 mmol/L of metformin, and 12 μg/ml of cisplatin. At 48 h and 72h, compared with blanks, the high dose group presented significant inhibition in cell division (*p* < 0.05). Also, the bevacizumab + cisplatin and bevacizumab + metformin + cisplatin groups were significantly more effective than any other treatment groups (*p* < 0.05). However, there was a significant difference in efficacies between the bevacizumab + cisplatin and bevacizumab + metformin + cisplatin groups (*p* < 0.05) **(** Figure 1E).

Comparative analysis between the low and high-dose treatments revealed that increased doses of the 2-drug regimen could gradually suppress the SKOV3 cell proliferation, while a similar increase in doses in the 3-drug regimen had a synergistic impact on the OC cells’ growth and survival (*p* < 0.01).

**FIGURE 2:**
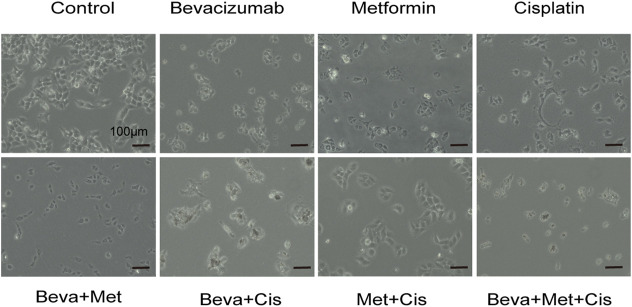
Morphological changes of SKOV3 cells treated with different combinations of drugs.

Different drug combinations significantly and differentially altered the morphology of SKOV3 cells. Among these treatment groups, the 3-drug combination of bevacizumab (2 mg/ml) + metformin (20 mmol/L) + cisplatin (12 μg/ml) significantly induced morphological changes in SKOV3 cells, resulting in the shrinkage, rounding and decrease in cell count, attenuated cell-cell adhesion and increased inter-cellular spacing, and decreased filopodia **(** Figure 2).

### *In vivo* effects of bevacizumab, metformin, and cisplatin combination on the growth of ovarian tumors

**FIGURE 3:**
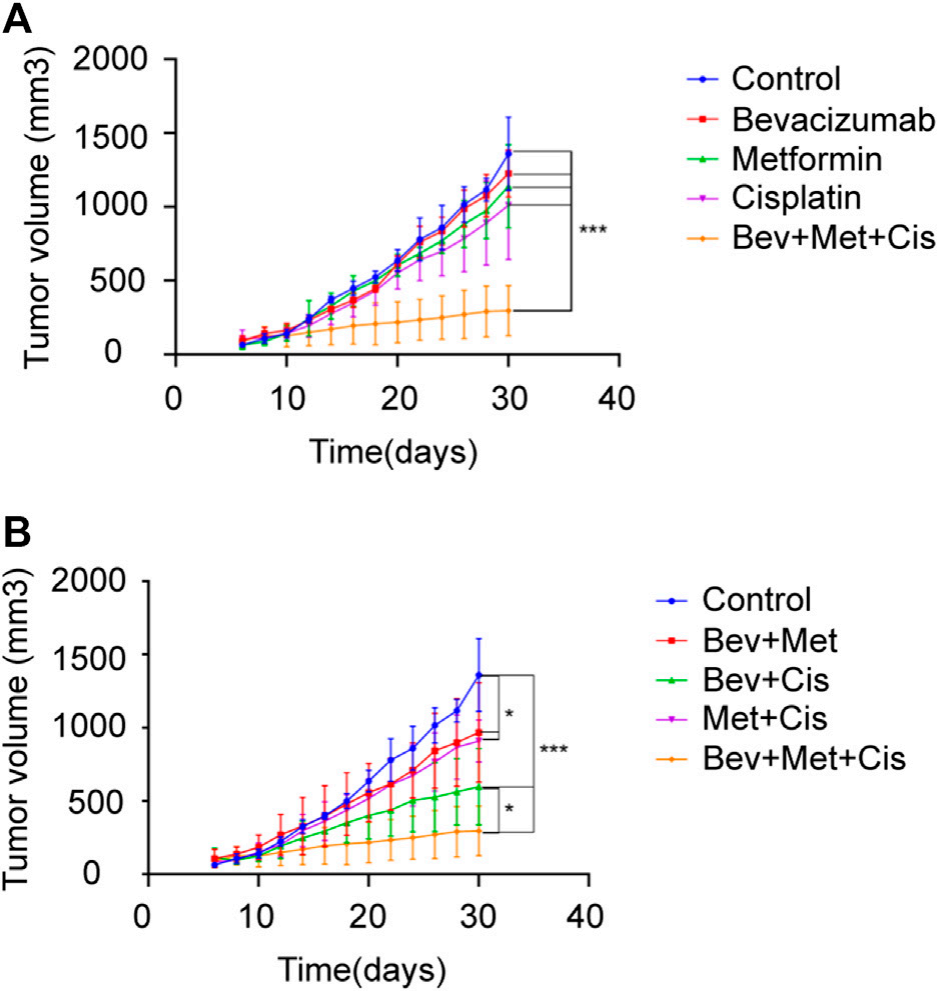
Effects of different drug combinations on the growth of SKOV3 xenografts. **(A)** Xenograft tumors in nude mice from each group. **(B–C)** Tumor growth curves of nude mice in each group after drug administration. *p < 0.05, **p < 0.01,***p < 0.001. The combination of bevacizumab and metformin reduces the population of CD44^+^/CD117^+^ CSCs.

Tumor-bearing nude mice were randomly divided into eight groups: A) blank controls, B) bevacizumab, C) metformin, D) cisplatin, E) bevacizumab + metformin, F) bevacizumab + cisplatin, G) metformin + cisplatin, and H) bevacizumab + metformin + cisplatin groups (Figure 3A).

Next, we evaluated the therapeutic effects of combined bevacizumab, metformin, and cisplatin with or without the growth rate of transplanted tumors of SKOV3 cells in nude mice. Compared with monotherapy, triple therapy significantly delayed the tumor growth (*p* < 0.05). Bevacizumab or metformin alone could not reduce the rate of tumor growth compared with that of the control group (*p* > 0.05). But cisplatin monotherapy slightly delayed the tumor growth (*p* = 0.059) (Figure 3B).

Notably, combination therapies showed overall tumor growth inhibition. Although both combinations of bevacizumab + metformin and metformin + cisplatin retarded the tumor growth rate compared with the blank control (*p* < 0.05), however, there was no significant difference in the degree of tumor inhibition between these two groups (*p* > 0.05). Together, these results suggest that 3-drug combination therapy can exert the maximum beneficial effects on the tumor growth inhibition in EOC patients (*p* < 0.001), followed by the dual therapy of bevacizumab + cisplatin (*p* < 0.001) (Figure 3C).

**FIGURE 4:**
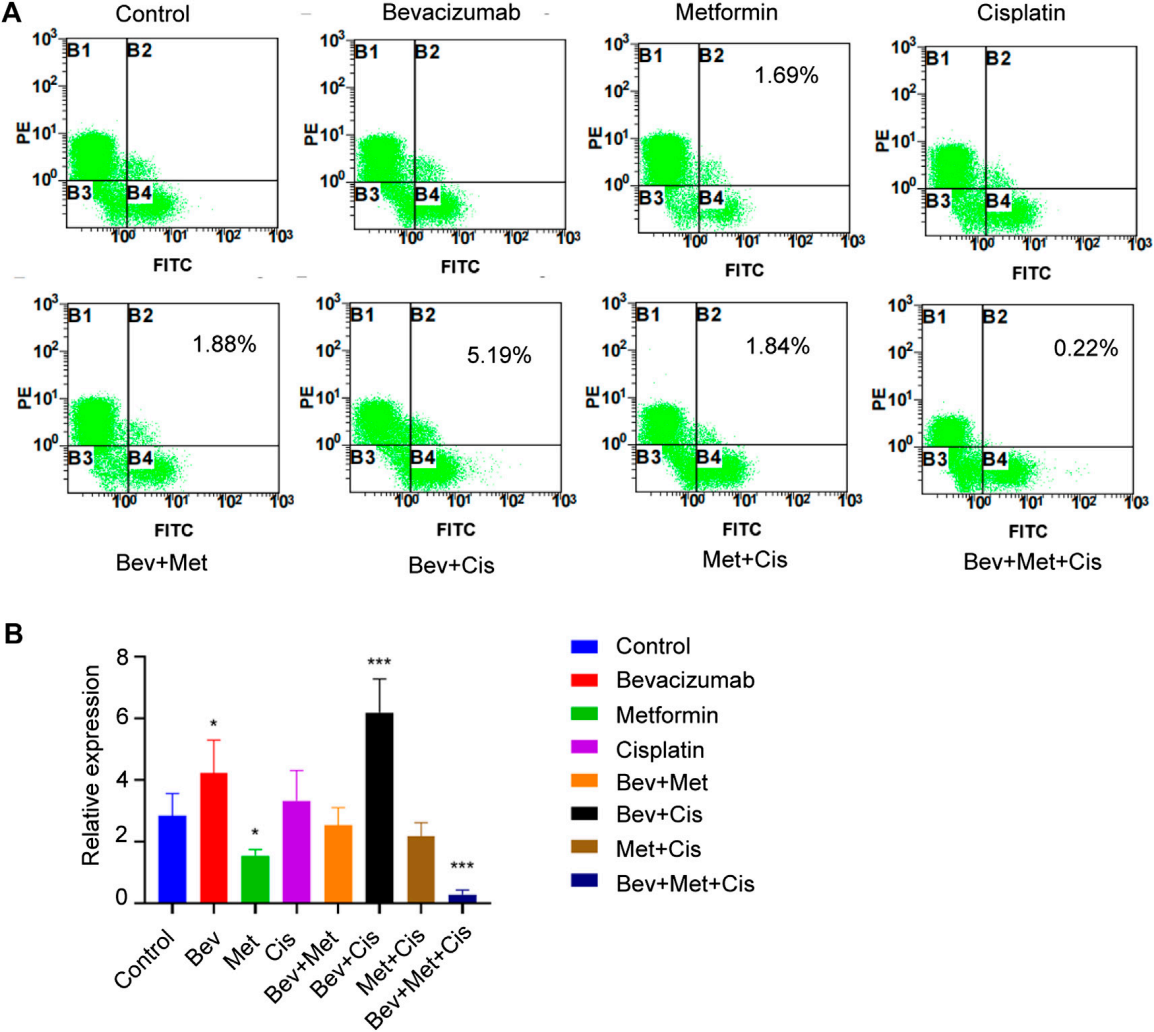
The combination of bevacizumab and metformin reduces the proportion of CD44^+^/CD117^+^ double-positive CSCs. **(A)** Proportion of CD44^+^/CD117^+^ double positive CSCs in each group. **(B)** Relative expressions of CD44^+^/CD117^+^ in each group.

Flow cytometric analysis was performed to evaluate the population of CD44^+^ and CD117^+^ CSCs to characterize their properties in the xenografts. Compared with the blank control group, percentages of CD44^+^/CD117^+^ dual-positive CSCs in the bevacizumab alone and bevacizumab + cisplatin groups were significantly increased (*p* < 0.05; *p* < 0.001, respectively). In contrast, metformin alone and 3-drug combination groups had downward trends (*p* < 0.05; *p* < 0.001, respectively). Interestingly, the proportion of CD44^+^ CSCs in nude mouse transplanted tumors was the lowest in the triple therapy group (Figures 4A,B).

### Expressions of VEGF, HIF-1α, and CD34 in xenograft tumors of nude mice

**FIGURE 5:**
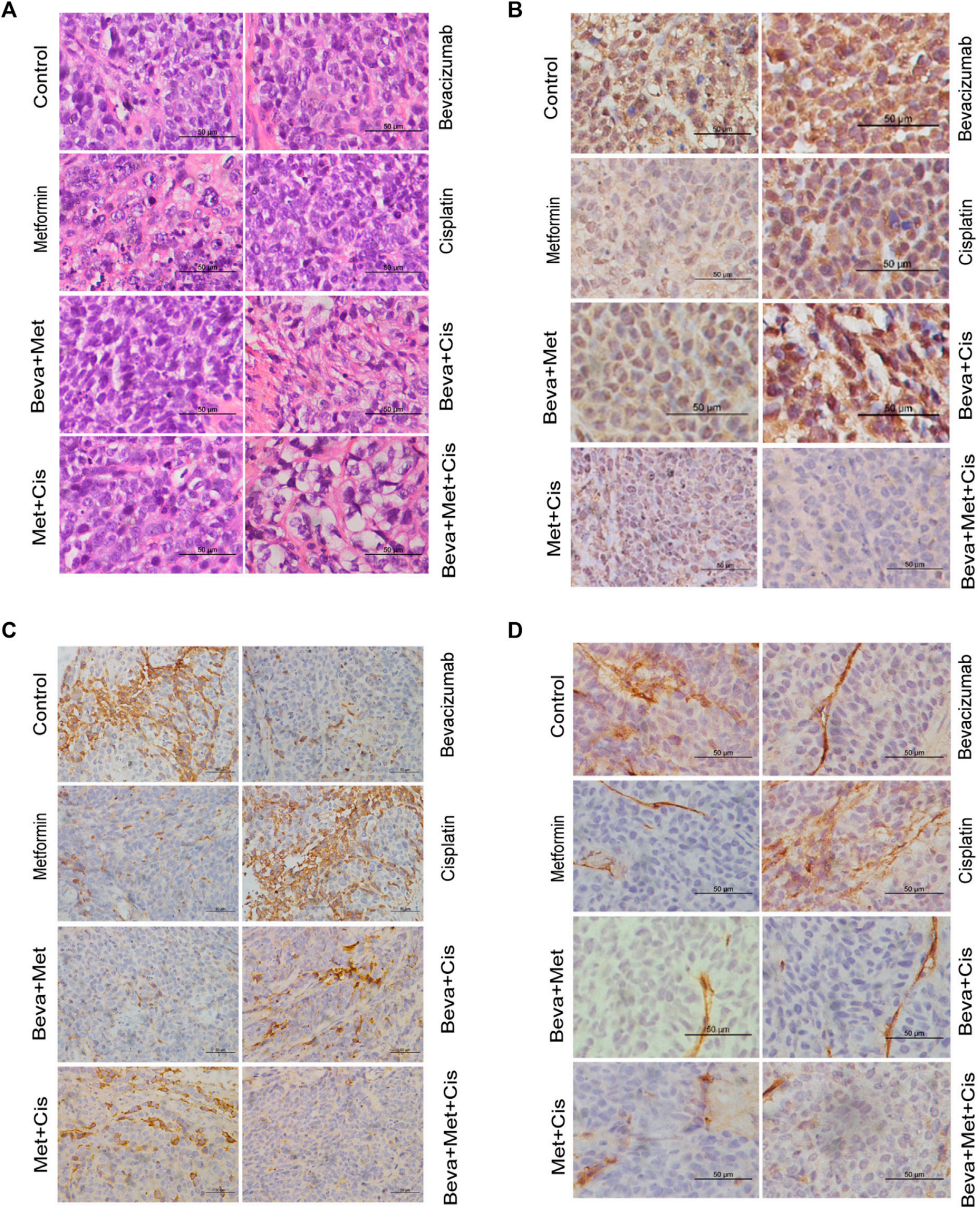
Effects of combinations of different drugs on the expression of HIF-1α, VEGF, and CD34 in transplanted tumors. **(A)** Transplanted tumor tissues of nude mice were subjected to the H&E staining (x400). **(B–D)** Immunohistochemistry (IHC) staining of HIF-1α **(B)**, VEGF **(C)**, and CD34 **(D)** in xenograft tumors (x400). **(E)** Comparison of the IHC scores of VEGF, HIF-1α, and CD34 in xenograft tumors of nude mice treated with different combinations of drugs. Note: Comparison of scores of experimental groups with that of the control groups (*p < 0.05, **p < 0.01, ***p < 0.001).

The H&E staining revealed indistinguishable cell boundaries, larger and intensely stained nuclei and the sign of atypia was obvious in each group. Tumor cells were seen as oval or round-shaped and diffused (Figure 5A).

MVD was evaluated by counting the number of CD34^+^ vascular endothelial cells. IHC analysis showed that expressions of VEGF and CD34 were mainly localized in the cytoplasm. HIF-1α was primarily located in the nucleus. Positive expression was indicated by the brownish yellow, brown, or tan-colored nuclei. The semi-quantitative integral method was used to score the disease progression as follows. 1) Proportion of positive cells: <10% was referred to 0 point; one point for 10–25%; 2 points for >25% but <75%; and three points for ≥75%. 2) Staining intensity: 0 points for colorless, one point for light yellow, 2 points for brown-yellow, and three points for tan color. The total score was obtained by summing up the scores from these two parameters. The scores of protein expression across the transplanted tumor tissues from each group were also compared.

All sections were independently assessed by two experienced pathologists. Whenever discrepancies arose, the section was evaluated by the third pathologist. The staining of VEGF, HIF-1α, and CD34 are shown in Figures 5B–D.

VEGF and CD34 expressions were significantly inhibited by the bevacizumab and metformin monotherapies compared with that of the control group (*p* = 0.001 and 0.006, respectively). Moreover, the bevacizumab + metformin and bevacizumab + metformin + cisplatin combination therapies efficiently downregulated the expression of these angiogenesis-related regulatory factors. Notably, VEGF and CD34 expressions were slightly different across the experimental groups (*p* = 0.001) (Figure 5E).

However, the HIF-1α expression was drastically up-regulated in the bevacizumab group. Compared with the control group, both HIF-1α and CD34 expressions were decreased in the metformin-treated group. Consistently, HIF-1α was decreased in mice that received the combination of metformin + bevacizumab + cisplatin compared with that of metformin alone treatment (Figure 5E). The reduction in tumor-specific expression of HIF-1α across the cisplatin, bevacizumab + metformin, bevacizumab + cisplatin, and metformin + cisplatin groups were not statistically significant compared with the control (Figure 5E).

These results suggest that the combination of metformin and bevacizumab can significantly reduce tumor angiogenesis and alleviate tumor hypoxia compared with either the monotherapy or blank control.

### Establishment of a limiting dilution assay for tumor recurrence

When the injected number of cells was adjusted to 10000/cell per nude mouse by the limit dilution method, recurrences were observed in the bevacizumab, bevacizumab + cisplatin, and bevacizumab + metformin + cisplatin treatment groups. But, no recurrence was observed in other groups, indicating that the recurrence rate did not decrease even with the inclusion of metformin (Table 1).

**TABLE 1:** Results of subcutaneous reseeding in nude mice in each group by limit dilution method.

Groups	Tumor formation (Inoculate 5000/10,000 cells)		Observation time (days)
	5000	10,000	
blank control	0/2	1/2	68
bevacizumab	0/2	1/2	70
metformin	0/2	0/2	-
cisplatin	0/2	0/2	-
bevacizumab + metformin	0/2	0/2	-
bevacizumab + cisplatin	0/2	1/2	50
metformin + cisplatin	0/2	0/2	-
bevacizumab + metformin + cisplatin	0/2	0/2	-

## Discussion

OC accounts for the highest mortality rate of all gynecological carcinomas posing a serious threat to women’s lives. Each year, more than 220,000 women are diagnosed with OC worldwide (Yue et al., 2021). Presently, 70% of patients who relapse within 3 years after the cytoreductive surgery combined with standard adjuvant chemotherapy mostly progress to chemo-resistance following repeated relapse, leading to death. With the advancement of precision medicine, targeted therapeutics such as angiogenesis inhibitors and poly (ADP-ribose) polymerase 1 (PARP-1) inhibitors have brought new promises in the treatment of OC patients. However, the tumor may also acquire chemo-resistant properties even with targeted therapies. Therefore, seeking solutions to drug resistance has become an important goal in resolving the poor efficacies of targeted agents. In this case, the combination of targeted anti-cancer and anti-CSCs regimens may have the clinical potential to overcome the hurdle of drug resistance and enhance anti-tumor activities.

Anti-angiogenesis therapy works by targeted inhibition of neovascularization in the TME by inducing the apoptosis in endothelial cells and blocking hematopoietic and endothelial cells from entering the neovascularization process. Bevacizumab, an inhibitor of VEGF, shows limited efficacy in OC (Guerrouahen et al., 2014). In this study, bevacizumab was combined with anti-CSC drug metformin to reveal that increasing doses of metformin could significantly block the proliferation of OC cells. Metformin combined with both bevacizumab and cisplatin also exhibited a synergistic tumor growth retardation effect 72 h post-treatment. These results provide a foundation for metformin combination therapy in OC treatment. To further confirm this finding, we used xenograft animal models of OC, which showed a very similar trend in tumor growth suppression and inhibition of CSC proliferation in the TME. Importantly, the tumor inhibition rate in the combination therapy was higher than that of the monotherapy group. The rate of inhibition was highest in the 3-drug regimen group than in the 2-drug, indicating that the inclusion of metformin remarkably enhanced the anti-tumor effects of bevacizumab and cisplatin.

Clinical and preclinical studies have shown that tumor cells can resist angiogenesis inhibitors through different mechanisms, resulting in poor clinical survival in OC patients. There are multiple possibilities by which OC cells can evade angiogenesis suppression effects, such as anti-angiogenic therapy-induced hypoxia may compensate for angiogenesis through other pro-angiogenic factors, thus making tumor cells resistant to treatments (Casanovas et al., 2005); anti-angiogenesis therapy can aggravate the degree of hypoxia, leading to proliferation and sequestration of chemo-resistant CSCs in the TME (De Bock et al., 2011), and anti-vascular therapy may enhance the ability of tumor cells to metastasize, thereby worsening the disease stage (Pàez-Ribes et al., 2009).

Hypoxia plays an important role in the development of several cancers. The transcription factor HIF-1α overexpression is found in the hypoxic TME. In the absence or under reduced oxygen levels, HIF-1α activates its target genes, like VEGF, to adjust the cellular metabolism and functions under hypoxia (Chen et al., 2015; Unwith et al., 2015). Here, expressions of VEGF and CD34 in the transplanted tumor tissues after bevacizumab treatment were lower, but HIF-1α expression was significantly higher than those in the control group, suggesting that the anti-angiogenic property of bevacizumab may exacerbate the hypoxic condition in the TME.

It has also been demonstrated that hypoxia can stabilize the stemness of CSCs in the TME (Eun et al., 2017). Tumor progression is a complex process, and cancer cells must under neoplastic changes to survive during the metastasis. Even the primary tumors are known to be heterogeneous, containing diverse subpopulations of cancer cells, allowing them to adapt to different tumor progression cascades. During this process, a small subpopulation of cancer cells develops stem-like properties in the tumor body to be called CSCs. CSCs play critical roles in disease progression because of their unique abilities to self-renew, be plastic, and differentiate as required. They can induce tumorigenesis, formation, or regeneration of heterogeneous cancer cell populations (Pinto et al., 2020). In addition to their high oncogenic potentials, CSCs are mostly resistant to currently available therapeutics, thus further promoting tumor metastasis and recurrence. CSCs are mainly identified by tumor-specific surface markers. The CD44^+^/CD117^+^ cells have the potential to be transformed into epithelial ovarian cancer (EOC) cell types (Zhang et al., 2008). We also widely exploited these markers to trace the differentiation of CSCs in the TME. We observed that bevacizumab treated transplanted tumors harbored increased proportions of CD44^+^/CD117^+^ cells in animal models. Furthermore, the level of HIF-1α expression detected increased in these cells, suggesting that hypoxia positively modulates the growth of CSCs.

Metformin was initially used for the treatment of diabetes. In recent years, several clinical investigations have identified that metformin can be repurposed to prevent metastasis and recurrence of various cancers, including OC (Chu et al., 2018; Mallik and Chowdhury, 2018). Its mechanism of action is well characterized. Hence, the inhibition of CSCs by metformin and the improvement of hypoxia in the TME is worthy of our attention (Saini and Yang, 2018; Meng et al., 2021). The findings of our study can improve the chemotherapeutic treatment strategies using targeted drugs (Markowska et al., 2017; Tang et al., 2021). Currently, there have been several ongoing trials testing the clinical efficacy of metformin inclusion in the standard chemotherapy or targeted therapy. Patil et al. (Patil, 2020) has found that metformin can alter the gene expression profile of primary oral CSCs. Cuyas et al. (Cuyàs et al., 2017) has confirmed that metformin can synergistically increase the sensitivity of breast CSCs to denosumab by inhibiting the overexpression of receptor activator of nuclear factor κB ligand (RANKL). Moreover, metformin can inhibit tumor growth and downregulate HIF-1α and VEGF expressions in gallbladder cancer (Ye et al., 2018). Metformin normalizes the expression of fibroblast growth factor 2 (FGF-2), reduces blood vessel density in obese mice, and restores tumor sensitivity to anti-VEGF therapies (Incio et al., 2018). A Phase-II trial in OC patients has demonstrated that metformin can increase the sensitivity of CSCs chemotherapy and is associated with an improved OS rate (Brown et al., 2020). In this study, both *in vitro* and *in vivo* experiments revealed that metformin inhibited OC cell proliferation, the proportion of CD44^+^/CD117^+^ CSCs, tumor growth, and HIF-1α expression. Levels of VEGF and CD34 were also decreased compared with the control group. In comparison to the controls, expressions of HIF-1α, VEGF, and CD34 were the lowest in the bevacizumab + metformin + cisplatin-treated cases, suggesting that metformin might change the hypoxia microenvironment induced by bevacizumab and inhibit vascular growth. Therefore, metformin combined with bevacizumab may be a promising anti-cancer regimen to overcome bevacizumab resistance and improve the prognosis in OC patients.

Flow cytometry was used to evaluate the effect of different drug combinations on the proportion of CD44^+^/CD117^+^ cells in xenografted nude mice. Results showed that the proportion of positive cells in the bevacizumab and cisplatin groups was significantly increased compared with the control group. However, the percentage of positive cells in the metformin group was decreased. Interestingly, the proportion of CD44^+^/CD117^+^ cells in the bevacizumab + metformin + cisplatin combination group was the lowest, which might be attributed to metformin’s ability to alleviate hypoxia in the TME and targeting CSCs, suggesting a potential synergistic effect of metformin when combined with bevacizumab and cisplatin.

Xenograft tumor cells treated with different drug combinations were subcutaneously transplanted into nude mice by the limit dilution method. It was found that only the bevacizumab, bevacizumab + cisplatin, and control groups had a recurrence. However, these results did not indicate that the addition of metformin reduced the recurrence rate. The possible reasons might include smaller sample size, shorter observation time, and a lower proportion of CSCs in the population. Fewer CSCs were retained after the limiting dilution, resulting in negative results for recurrence. Comprehensive analyses of flow cytometry and immunohistochemistry results revealed that bevacizumab treatment enhanced the HIF-1α expression and increased the proportion of CD44^+^/CD117^+^ cells, resulting in the tumor recurrence in both the bevacizumab alone and bevacizumab + cisplatin groups. But the effect of metformin, including 3-drug therapy, on recurrence rate was not identified. Therefore, the influence of different drug combinations on the recurrence rate needs to be further investigated.

The emergence of CSC theory provides a new direction for the treatment of OC patients. Targeting and eliminating CSCs are of great significance for the effective treatment of cancers, including OC. During tumorigenesis, CSCs play a vital role in conferring chemo-resistance, recurrence, and metastasis. Through interaction with cytokines, a hypoxic TME is established providing a favorable environment for the generation and maintenance of CSCs. And anti-angiogenic drugs accelerate this process. Therefore, the combined administration of anti-angiogenic and anti-CSCs agents would effectively reduce the chances of chemo-resistance-associated sudden relapse and metastasis in OC patients. On the other hand, metformin, as a first-line drug for the treatment of type-2 diabetes, is suitable for rapid clinical application in the treatment of OC due to its established safety, low cost, and targeted suppression of CSCs as well. In summary, this study provided new ideas for the cost-effective treatment of OC, improved rates of progression-free survival rate and OS, and prognosis of patients.

There are still some limitations of this study that need to be addressed. First, a large number of literatures suggest that CD44, CD117, CD133, CD24, aldehyde dehydrogenase (ALDH) and other markers can be used to investigate cancer stem cells in certain ovarian cancer types. We only use CD44/CD117 as markers of ovarian cancer stem cells in this study, lack of research on the expression of CD133 and ALDH. Second, this study is only a preliminary exploration of metformin combined with other antitumor drugs. The experimental results are based on *in vitro* cell studies and tumor animal models. The experimental method is relatively simple, and other methods can be added to enhance the scientific nature of the study. For example, the sphere formation assay of tumor stem cells can be used to measure the self-renewal ability and migration mobility of tumor cells. Third, xenograft model was not the best animal mode, and during the treatment evaluation, only one measurement was used, which is tumor size. Therefore, it is necessary to add sphere formation assay to evaluate the sphere formation ability of ovarian cancer cells, to add markers of tumor stem cells, and to use multiple treatment evaluation methods to evaluate the efficacy of treatment.

## Conclusion

In this study, bevacizumab treatment aggravated the degree of hypoxia in the TME, induced overexpression of HIF-1α, and increased the proportion of CD44+/CD117+ CSCs. While metformin could alleviate the hypoxic condition in the TME inhibiting the growth of CD44+/CD117+ CSCs. Metformin combined with bevacizumab and cisplatin inhibits the growth of human ovarian cancer SKOV3 cells *in vitro* and *in vivo*, which may be a reasonable and promising treatment option for improving the prognosis of OC patients.

## Data Availability

The original contributions presented in the study are included in the article/Supplementary Material, further inquiries can be directed to the corresponding authors.
