# Supplementary figures and images for: Metformin anticancer: Reverses tumor hypoxia induced by bevacizumab and reduces the expression of cancer stem cell markers CD44/CD117 in human ovarian cancer SKOV3 cells
Source: Front Pharmacol. 2022 Aug 15;13:955984. doi: 10.3389/fphar.2022.955984 (PMC9421358; doi:10.3389/fphar.2022.955984)

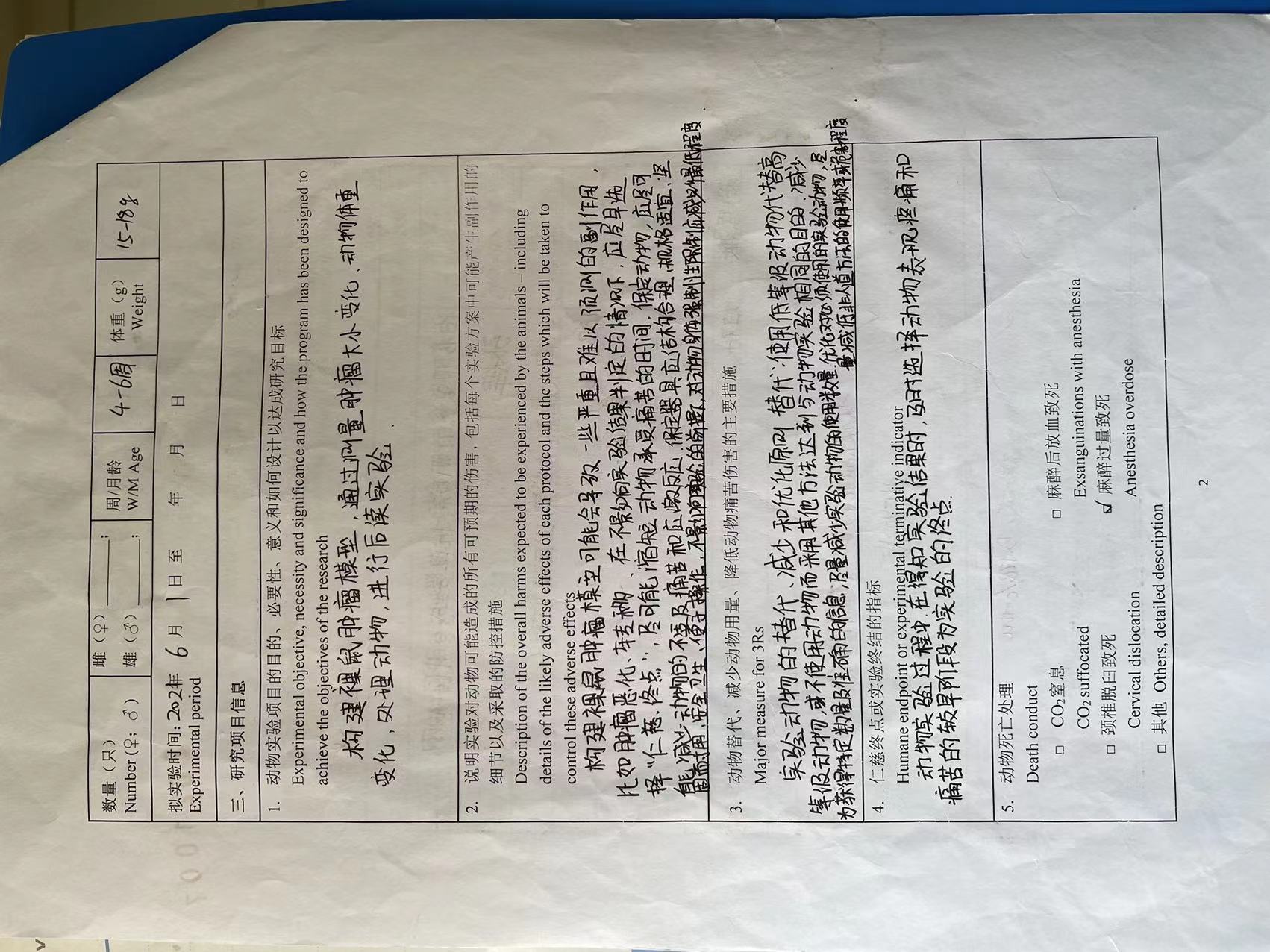

Supplement: Supplementary file 1 [file DataSheet1.ZIP › ┬╫└φ▒φ2.jpg]

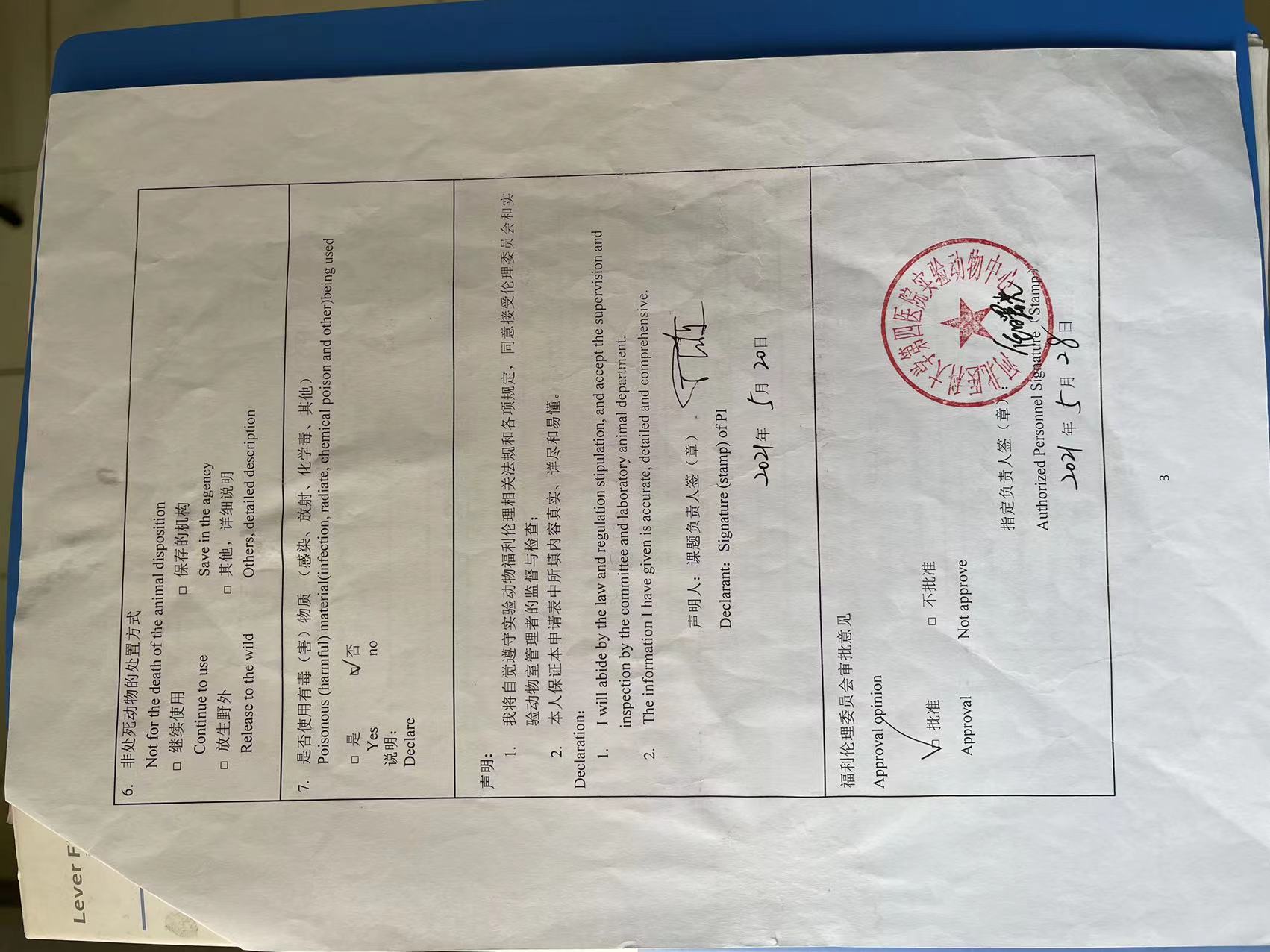

Supplement: Supplementary file 1 [file DataSheet1.ZIP › ┬╫└φ▒φ3.jpg]

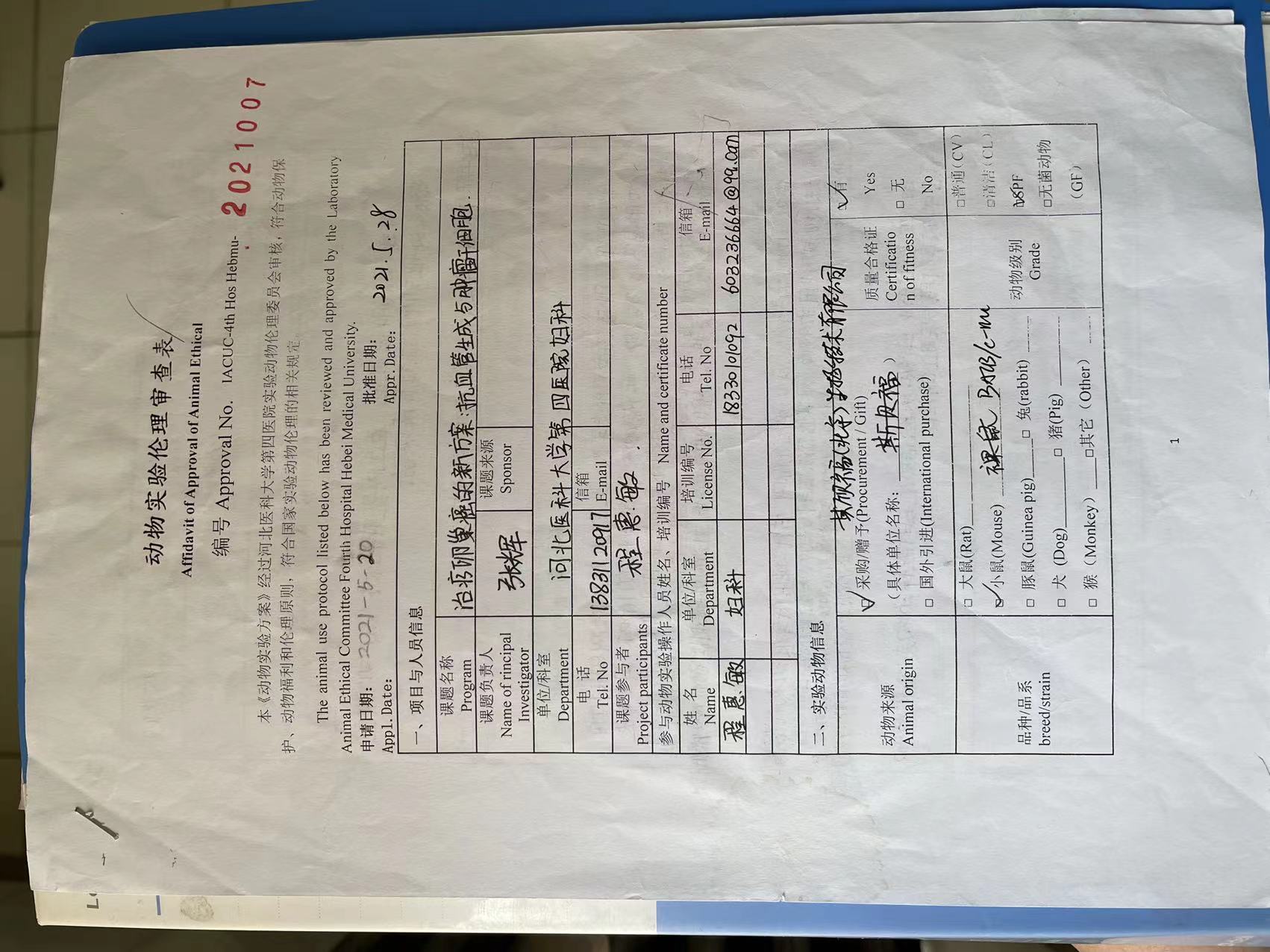

Supplement: Supplementary file 1 [file DataSheet1.ZIP › ┬╫└φ▒φ1.jpg]

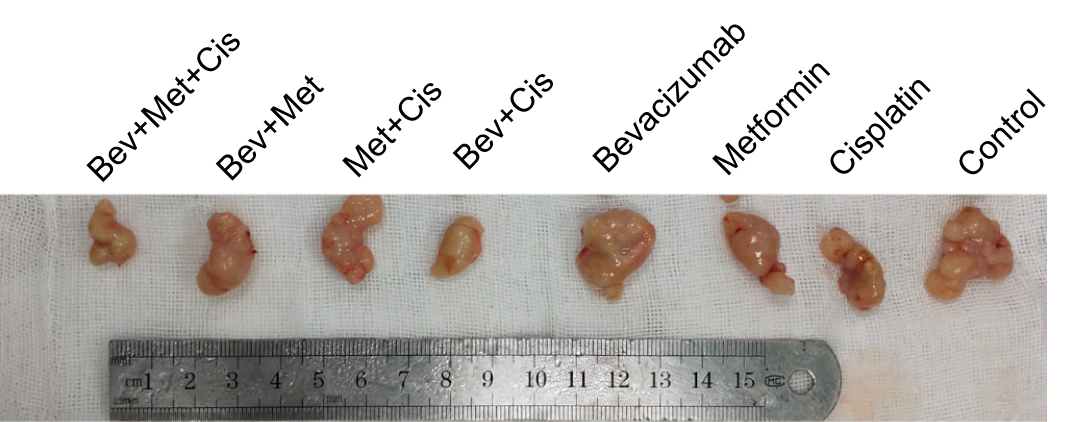

Supplement: Supplementary file 2 [file Image2.tif]

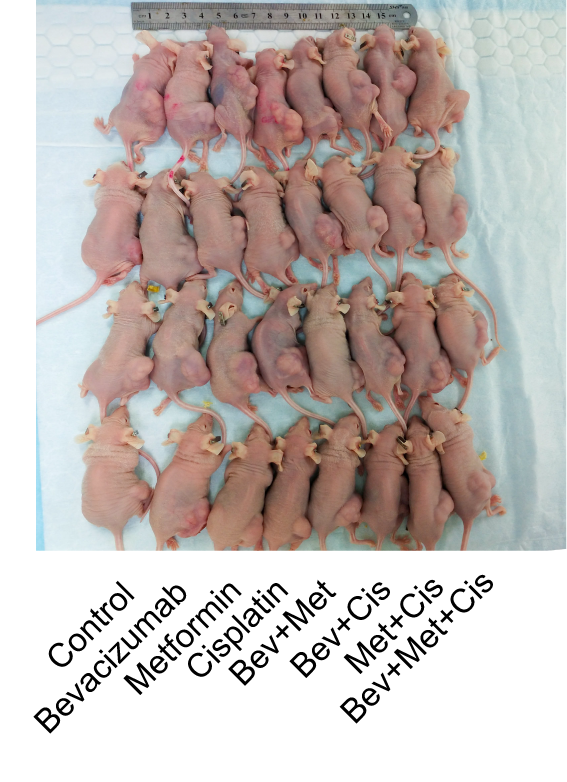

Supplement: Supplementary file 3 [file Image1.tif]
