# Supplementary material for: Metformin anticancer: Reverses tumor hypoxia induced by bevacizumab and reduces the expression of cancer stem cell markers CD44/CD117 in human ovarian cancer SKOV3 cells
Source: Front Pharmacol. 2022 Aug 15;13:955984. doi: 10.3389/fphar.2022.955984 (PMC9421358; doi:10.3389/fphar.2022.955984)
Supplement: Supplementary file 4 [file Table2.DOCX]

| Groups | Tumor formation  (Inoculate 5000/10000 cells) | | Observation time (days) |
| --- | --- | --- | --- |
|  | 5000 | 10000 |  |
| blank control | 0/2 | 1/2 | 68 |
| bevacizumab | 0/2 | 1/2 | 70 |
| metformin | 0/2 | 0/2 | - |
| cisplatin | 0/2 | 0/2 | - |
| bevacizumab+metformin | 0/2 | 0/2 | - |
| bevacizumab+cisplatin | 0/2 | 1/2 | 50 |
| metformin+cisplatin | 0/2 | 0/2 | - |
| bevacizumab+metformin+cisplatin | 0/2 | 0/2 | - |
